# A Delay-Aware and Reliable Data Aggregation for Cyber-Physical Sensing

**DOI:** 10.3390/s17020395

**Published:** 2017-02-17

**Authors:** Jinhuan Zhang, Jun Long, Chengyuan Zhang, Guihu Zhao

**Affiliations:** School of Information Science and Engineering, Central South University, Changsha 410083, China; jinhuan_zhang@csu.edu.cn (J.Z.); cyzhang@csu.edu.cn (C.Z.); guihuzhao.csu@gmail.com (G.Z.)

**Keywords:** physical sensing, reliability, delay, tiered structure routing

## Abstract

Physical information sensed by various sensors in a cyber-physical system should be collected for further operation. In many applications, data aggregation should take reliability and delay into consideration. To address these problems, a novel Tiered Structure Routing-based Delay-Aware and Reliable Data Aggregation scheme named TSR-DARDA for spherical physical objects is proposed. By dividing the spherical network constructed by dispersed sensor nodes into circular tiers with specifically designed widths and cells, TSTR-DARDA tries to enable as many nodes as possible to transmit data simultaneously. In order to ensure transmission reliability, lost packets are retransmitted. Moreover, to minimize the latency while maintaining reliability for data collection, in-network aggregation and broadcast techniques are adopted to deal with the transmission between data collecting nodes in the outer layer and their parent data collecting nodes in the inner layer. Thus, the optimization problem is transformed to minimize the delay under reliability constraints by controlling the system parameters. To demonstrate the effectiveness of the proposed scheme, we have conducted extensive theoretical analysis and comparisons to evaluate the performance of TSR-DARDA. The analysis and simulations show that TSR-DARDA leads to lower delay with reliability satisfaction.

## 1. Introduction

Sensing is one of the essential components of a cyber-physical system (CPS). Different types of sensors are being deployed to collect a large amount of sensing data about the environment (temperature, humidity), transportation, terminals, and even social activities. Wireless sensor networks (WSNs) are an essential way to realize CPS sensing. Wireless sensor networks have been widely recognized as a ubiquitous and general approach with extensive applications in agriculture, industry, and unattended environments, such as habitat monitoring, surveillance and tracking for the military. Wireless sensor networks can provide industrial, agricultural, medical and vehicular Internet of Things or CPS service. They also make smart factories, e-healthcare and intelligent transportation real. Wireless sensor networks consist of a large number of low-cost and low-power sensor nodes that obtain information of physical objects, which can collaborate with each other to collect data and disseminate it to sinks (or gateway nodes) through an established routing path [1,2]. Due to the fact they provide fine-grained spatial-temporal sensing, communication and computation at a low premium of cost and power, wireless sensor networks are a way to support CPS. 

**Figure 1:**
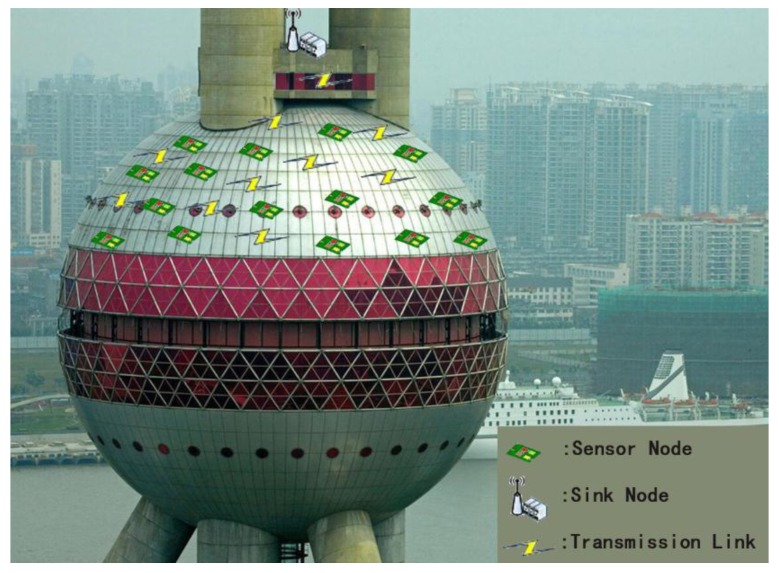
Sensor nodes deployed to collect information for a TV tower.

In the paper, we focus on the surface information collection of spherical objects in CPS such as machinery, buildings (where sensor nodes are deployed on a TV tower to collect information as shown in Figure 1), etc. In the data collection procedure, time is slotted. In order to avoid transmission interference, nodes are assigned different time slots to complete data packet processing and transmission. Nodes without interference could transmit simultaneously. Besides, multi-hop communication is used to relay the information from the source node to the sink for further data management or operation. In order to achieve significant performance improvements in energy consumption, memory usage, bandwidth, and delay, distributed in-network aggregation is applied to improve the communication efficiency of the system [3,4]. 

Meanwhile, in wireless communication, due to the complexity of the transmission channel and the harsh environment, reliable communications are essential for most WSNs applications because of the unreliable links [5,6,7,8,9,10]. Although many applications require that each data packet be successfully delivered to sink with statistical probability δ < 1, such as 60%–95%, some reports reveal that the wireless link packet loss rate may be up to 70% in real WSNs, which is far from being satisfactory [11,12,13]. Thus, it is more important to ensure reliability in communication.

Moreover, in WSNs, delays also play an important role [14]. For data collection, delay is generally defined as the sensed data transmission time from source nodes to sink, named transport delay or end-to-end (E2E) delay [15]. For information processes, being beyond of the time limit may cause information loss or affect the next series of data processing. Besides, in many safety-critical industry applications, missing urgent information may cause severe property loss and casualties, which are often not acceptable. Therefore, in order to rapidly respond to the event and satisfy an acceptable delay, the detected information should be transported quickly to the sink for further manipulation. 

Consequently, the delay for data collection and data transmission reliability should be considered in data collection of CPS. To address these issues, a novel delay-aware and reliable data aggregation scheme based on tiered structure routing, named Tiered Structure Routing-based Delay-aware and Reliable Data Aggregation (TSR-DARDA), is proposed in this paper for spherical physical objects. TSR-DARDA takes both data reliability and transport delays into consideration. Our main contributions can be summarized as follows:

(1) A novel TSR-DARDA scheme is proposed to achieve delay performance subject to reliability constraint for data collection of spherical physical objects. Since many applications have both reliability and delay constraints, the proposed scheme named TSR-DARDA targets both optimization of reliability and network delay. Compared with the existing data collection schemes, the important differences among them are that TSR-DARDA would partition the surface of a spherical network into circular tiers with carefully designed widths and it could meet the goal that when data in the outer layer finishes transmission broadcasting to parent nodes in the inner layer, the nodes in the inner layer just finish data aggregation, thus minimizing the latency while maintaining data collection reliability.
(2) The distributed in-network aggregation technology adopted in TSR-DARDA scheme can effectively further minimize data collection latency in WSNs for spherical physical objects. It is challenging to reduce delay with the precondition of ensuring reliability. In this paper, the data aggregation delay is improved through the careful design of surface tiers. Moreover, each surface tier is further partitioned into cells. Those cells that do not interfere with each other could simultaneously finish data aggregation by unicast and retransmission within each cell. Therefore, the network delay optimization is obtained under the precondition of guaranteed reliability.
(3) The selection of parameters in TSR-DARDA scheme is discussed to satisfy the surface tier partition optimization. An algorithm is presented to describe the detailed procedure to select the optimizing parameters, which can ensure the reduction of transport delay without reducing the data reliability. In addition, theoretical analysis and comprehensive simulation experiments are conducted to verify the effectiveness of the TSR-DARDA scheme. The results show that the proposed TSR-DARDA could obtain the goal of optimization of network delay subject to network reliability.

The rest of this paper is organized as follows: Section 2 reviews related works. We formally define the problem and the system model in Section 3. Section 4 presents TSR-DARDA scheme, including parameter selection optimization and performance analysis. Performance evaluations through simulations are presented in Section 5. Section 6 concludes the paper.

## 2. Related Work

Sensing is a key component of cyber-physical sensing. Meanwhile, data collection is a key function of sensing. Various kinds of data sensed from cyber-physical objects including machines, equipment or vehicles need to be collected to gain valuable information for further processing and operation, such as machine control, failure prediction, and so forth. To support cyber-physical sensing, wireless sensor networks are frequently applied. Collecting the gathered information efficiently is a key issue for wireless sensor networks. A great deal of work has been devoted to this field [16,17,18,19,20,21]. Li et al. [22] studied the time complexity, message complexity, and energy cost complexity of some data collection, data aggregation, and queries for a multi-hop wireless sensor network of *n* nodes. Network delay and reliable communication have an important influence on WSN applications and need to be considered in many WSN studies. There are many research efforts devoted to providing a reliable transmission service because of the unreliable links in WSNs [7,8,9,23,24,25]. Liu et al. [9] pointed out that there are mainly two categories, including packet-loss avoidance and packet-loss recovery. Packet-loss avoidance (e.g., [12]) attempts to reduce the occurrence of packet loss and packet-loss recovery (e.g., [25]) tries to recover from the packet loss when it happens. Because packet-loss avoidance methods need to pay a high price and from the cost consideration the most widely used mechanism in networks is based on packet-loss recovery.

The tiered structure has appeared in the literature for Low Energy Adaptive Tier Clustering Hierarchy [26]. In the scheme, a two-level hierarchical approach has been proposed to organize a sensor network into a set of clusters and every cluster is divided into smaller clusters called mini clusters. As the way the clusters are organized, for each mini cluster a mini cluster-head is defined. The mini cluster-head communicates with the cluster-head directly, and it aggregates its mini-cluster information. Joo et al. [3] developed a new network with tiered structure for in-network computation for a class of generalized maximum functions, which focuses on the delay performance of the function computation subject to reliability constraints in lossy wireless environments. They showed that aggregation using wireless broadcast technology can greatly reduce the delay while meeting the reliability requirement. Zhang et al. [27] suggested a novel variable width tiered structure routing (VWTSR) scheme, wherein the network is divided into circular tiers with different widths and each tier is further partitioned into cells. Those cells that do not interfere with each other could simultaneously finish data aggregation by unicast and retransmission within each cell. 

However, the proposed VWTSR scheme is suitable for flat networks. Moreover, inspired by [28], which has proposed an energy-efficient and delay-aware wireless computing system to satisfy an acceptable delay and low power consumption for data collection, the main focus of the paper is to study the efficient data collection scheme taking reliability and delay into consideration for cyber-physical sensing with spherical networks.

## 3. The System Model and Problem Statement

### 3.1. The System Model

**Figure 2:**
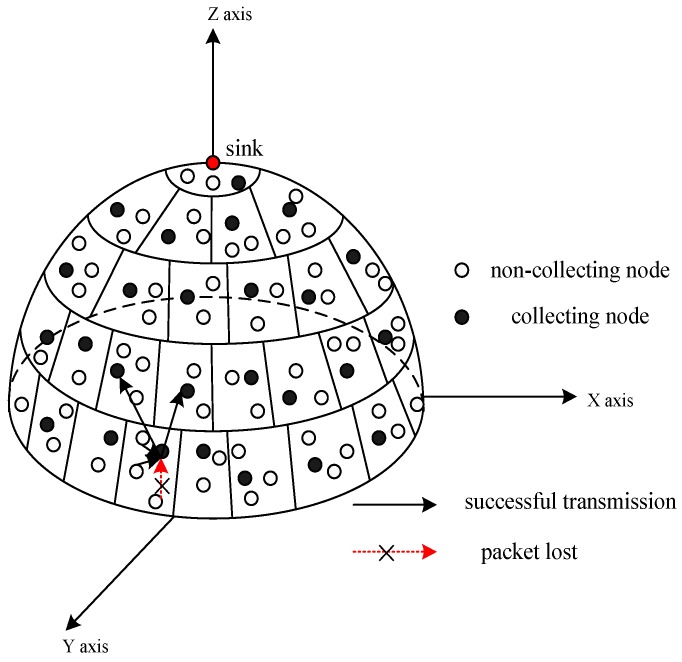
The envisioned spherical network with sensors deployed in physical objects.

Consider an envisioned wireless sensor network consisting of sensor nodes that are uniformly and randomly scattered in the surface of an half sphere, which are shown in Figure 2. The radius of the half sphere is *R* (m) and the node density is *ρ* (m^−2^). Furthermore, nodes do not move after being deployed. In a data gathering round, each sensor node generates a packet, whose payload includes sensed data without error and loss. The sensor nodes consist of two types of nodes, non-collecting and collecting nodes. Each non-collecting sensor node only generates its own data and forwards it to the corresponding collecting nodes by unicast. However, each collecting node not only senses its own data, but also aggregates the data from other nodes and forwards them to the sink through multi-hop wireless communications by broadcast. In the multi-hop transmission, routing is fixed. Besides, time is divided into slots. Each node is assigned the time slot to ensure transmission without collision. The system model assumes that all sensor nodes are synchronized, and the packet loss rate during the wireless channel transmission is 1 − *p* for all links. In order to avoid the information loss caused by packet lost, a retransmission technique is applied. 

Furthermore, there is the following assumption:

**Assumption** **1.** Each non-collecting node generates its own data, and forwards it to the corresponding collecting nodes with a small identical transmission range, which is denoted by ℜ. In order to forward data in the outer tier to its parent collecting nodes in the inner tier, each collecting node has the same transmission power corresponding to the larger transmission range r_o_. The nodes’ interference radius is 2r. The gateway or sink node, which collects information and sends the collected information to the user terminal by Internet or WiFi, has one wireless channel. To avoid transmission interference, the wireless channels between two adjacent tiers are independent across links. Moreover, for each collecting node in adjacent inner tier, there is one father collecting node in hotspots, wherein the shortest distance to the sink is less than r. Nodes in hot-spots transmit data directly to the sink.

### 3.2. The Data Aggregation Model

In the data collection process, an intermediate or collecting node can collect information from other sensor nodes. Then, it aggregates them into a unit of information, i.e., a packet, and then forwards the computed and aggregated packet to the next hop or to the sink. In this paper, data aggregation refers to the situation in which several data packets meet at a node in the routing procedure and they are aggregated into one new data packet. The new data packet has the same size with the original data packet.

The lossless step-by-step multi-hop aggregation model is adopted to do data aggregation [2]. In the data aggregation model, the aggregation of node *i* with its *χ* multiple inputs is implemented in an orderly way, that is, receiving data is aggregated with current data according to the arrival order. Let *υ_i_* represent the source data packet of node *i*, and ϑ(i,j) represents the intermediate temporary aggregation result of node *i* and node *j*. For simplification, $\vartheta_{i}$ denotes the current temporary aggregation result of node *i*. We apply $\psi_{i}$ to denote the final aggregation result of node *i* with all receiving nodes’ data and its own data.

When data $\vartheta_{j}$ from node *j* is transmitted to node *i*, node *i* aggregates $\vartheta_{j}$ with its own data (may be the origin data *υ_i_* or the intermediate temporary data $\vartheta_{i}$). If current data packet of node *i* is the origin data *υ_i_*, and data from *j* is also origin data $\vartheta_{j}=\upsilon_{j}$, which means that the data to be aggregated is both origin source data, the aggregation formula is:

(1) $$\vartheta (i,j)=\varepsilon \max(\upsilon_{i},\upsilon_{j})+(1-\varepsilon )\min(\upsilon_{i},\upsilon_{j})$$

where, min and max denote the class of general minimum and maximum computation functions. In Formula (1), ε is the correlation coefficient and the value is between 0 and 1. When ε = 1 and ε = 0, it respectively represents the class of general maximum and minimum computation functions. When doing aggregation, if any one of to be aggregated data is not origin source data, the aggregation formula is as the follows:

(2) $$\vartheta (\vartheta_{i},\psi_{j})=\mu \max(\vartheta_{i},\psi_{j})+(1-\mu )\min(\vartheta_{i},\psi_{j})$$

In Formula (2), μ denotes the impact factor and it is a decimal not bigger than 1 (e.g., μ = 0.8). Min and max denote the class of general minimum and maximum computation functions. $\vartheta_{i}$ and $\psi_{j}$ respectively refer to the intermediate temporary aggregation result of node *i* and final aggregation result of child node *j*. There is at least one non-origin data packet in $\vartheta_{i}$ and $\psi_{j}$.

### 3.3. The Network Delay Model

End-to-end (E2E) delay is defined as the time from a packet’s first transmission until its successful arrival at the sink. Let $\Gamma_{i}$ denote the end-to-end delay from node i to sink. We define the network delay as the maximum time required for all nodes in the network finishes its single transmission to the sink. Let D denote the network delay.

Following the network delay model in [25], assume that there are h hops from the source node i to the sink. Let $P_{i}$ denote one routing path from node i to the sink, where $P_{i}$ = {$v_{0}^{i}$, $v_{1}^{i}$, *…*, $v_{h}^{i}$}, $v_{h}^{i}$ denotes the node whose distance to the sink is h hops in the routing path of node *i*. The delay at each hop caused by transmission and aggregation processing are denoted by $\tau_{i}^{T}$ *=* [$\tau_{0}$, $\tau_{1}$, *…*, $\tau_{h}$]. Therefore, the transport E2E delay of packet transmitted to sink from node *i* is $\Gamma_{i}$ = $\sum_{k=0}^{h}\tau_{k}$. For the distributed in-network aggregation we have the equation $D=\max\{\Gamma_{i}\}\;(i=1,2,3,...)$.

### 3.4. Problem Statement

In this part, some definitions are given firstly to ensure clear statements. They are as follows:

**Definition** **1.** *Transmission Reliability. It represents the statistical probability of packets successfully forwarded to sink from a node in QoS level. Let $\delta_{i}$ denote the transmission reliability of packets transmitted from node i to a sink.*

**Definition** **2.** *Network Reliability. It represents the statistical probability of packets successfully forwarded to the sink from all nodes in QoS level. Each sensed data is received by the sink with a probability not less than δ, where δ represents the network transmission success rate constraint.*

As stated in the system model, the information sensed by an individual non-collecting node is collected by some special colleting nodes, and then these collecting nodes are responsible for data aggregation and information forwarding to the sink. Each collecting node at depth *d* or tier *d* has at least *x* (*x* ≥ 1) collecting parents at depth or tier *d* − 1 (except the hotspots and there is only one parent collecting node in hotspots). It transmits the packet through the wireless broadcast channel to its parents (1 + *μ*) times, where, *μ* denotes the number of retransmission times. We say that a node successfully transmits a packet if the broadcast packet is successfully received by one of *x* parents. In the meantime, we try to minimize network delay.

The focus of the paper is to improve the network delay performance while achieving the same level of network reliability. That is *δ_i_* ≥ *δ*. To sum up the above, the goal can be expressed as the following expression, that is, as for any node *i* in the network it meets the following formula:

(3) $$\{\begin{array}{c} \min(D)=\min\underset{0<i\leq n}{\max(\Gamma_{i})}\\ \Gamma_{i}=\sum_{k=0}^{h}\tau_{k}\\ s.t.\;\delta_{i}\geq \delta \end{array}$$

Hence, the target is minimizing the maximum transport E2E delay under the guarantee of transport service quality, e.g., *δ_i_* ≥ *δ*.

## 4. Design of the TSR-DARDA Scheme

In the section, the proposed TSR-DARDA scheme is described. As an illustration of the methods, the idea and implementation of TSR-DARDA scheme is presented in detail. Then, how to select parameters to satisfy the optimizing requirements is discussed. At the end of the section, we conduct a theoretical analysis on the delay performance.

### 4.1. The Overall Approach

**Figure 3:**
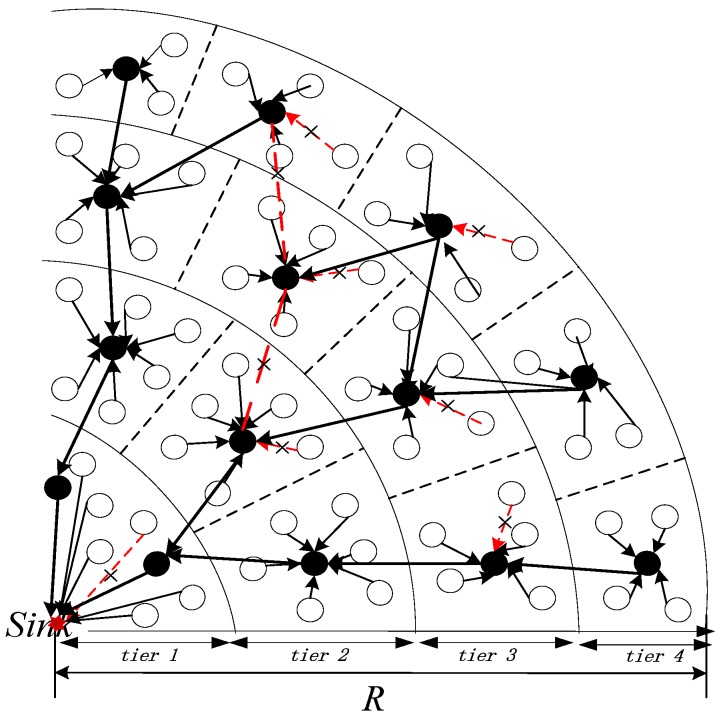
The overall approach.

In this section, the overall TSR-DARDA approach is described. To ensure a clear statement, the system model shown in Figure 2 is vertically projected on to the plane from top to bottom, which is shown in Figure 3. As illustrated in Figure 3, the surface is partitioned into circular tiers and each tier is further divided into cells. Non-collecting nodes transmit data packet to collecting nodes in each cell. Those cells that do not interfere with each other could simultaneously aggregate data by unicast and retransmission within each cell. If the packet is lost in the transmission, it will be retransmitted until the maximum number of retransmissions. Moreover, in the surface circular tier partition, the tier width should meet the goal that the collecting nodes in the inner layer just finish data aggregation when the collecting nodes in the outer layer finish transmission to parent collecting nodes in the inner layer, thus minimizing the latency while maintaining reliability for data collection. All the data will be gradually aggregated to a sink by the distributed in-network aggregation under the assumption described in the system model that the wireless channels between two adjacent tiers are independent across links.

Therefore, the proposed scheme consists of two data collection phases: (1) at one time slot, each non-collecting node in those cells free of interferences transmits its packet to its parent collecting node in the same cell simultaneously and (2) each collecting node receives the packet and implements aggregation. After data aggregation of the cell is finished, it transmits the aggregated packet to its parent collecting nodes in the inner tier in the same time slot with other nodes without transmission interference. The data collection is implemented simultaneously and the aggregated data is forwarded tier by tier from the outermost tier to the sink node.

### 4.2. TSR-DARDA Scheme

In the scheme, the key point is how to partition the surface of the sphere into circular tiers with careful designed widths and how each tier is further divided into cells except for the hotspots, as shown in Figure 1. In the following, more details will be given on the key points and their implementation.

#### 4.2.1. Circular Tier and Cell Partitioning of the Surface of the Half Sphere

**Figure 4:**
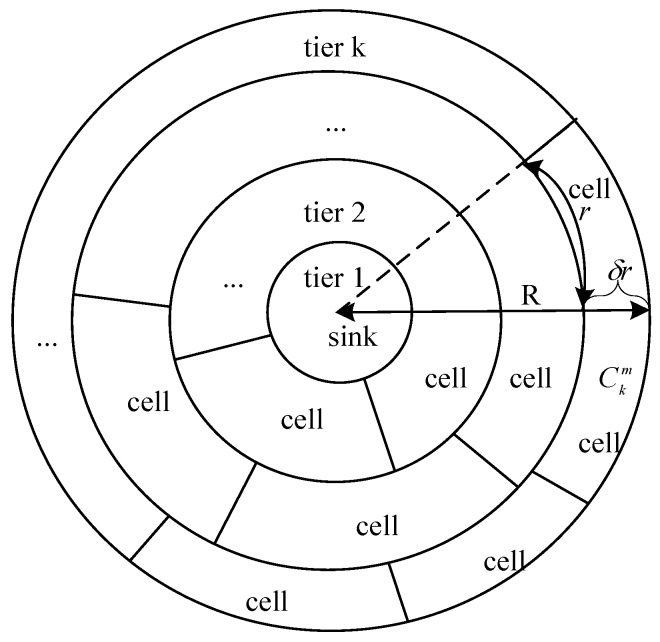
Projection from the three-dimensional space to two-dimensional plane of tiers and cells partition.

For the envisioned half sphere as described in Section 3.1, the sink is located at the spherical vertex and assume nodes’ interference radius is 2*r*. To ensure a clear statement, the three-dimensional circular tier and cell partitions are projected onto a two-dimensional plane from top to bottom as shown in Figure 4. The width of each tier is firstly calculated according to the constraint that the collecting nodes in the inner layer just finish data aggregation when the collecting nodes in the outer layer finish transmission to the parent collecting nodes in the inner layer. That means $\tau_{k-1\_n}=\tau_{k}$ and $\tau_{k}=\tau_{k\_n}+\tau_{k\_g}$, where $\tau_{k}$ represents the data aggregation time needed for tier *k* and it equals the sum of time required by non-collecting nodes aggregates data to collecting nodes, which denoted by $\tau_{k\_n}$, and time of collecting nodes aggregates data to parent collecting nodes in inner tier, which denoted by $\tau_{k\_g}$. $\tau_{k-1\_n}$ represents the time needed for tier k − 1 to finish non-collecting nodes data aggregation to collecting nodes in the same tier. Accordingly, the surface could be divided into circular tiers $\left\{T_{k}\right\}$.

Assume the width of the outmost tier $T_{k}$ is δr. That is $d_{k}=\delta r$ (the value of δ will be discussed in following Discussion section). It is partitioned into cells according to the width of *r* at the boundary of the inner tier as shown in Figure 4. The *m*-th cell in *k* tier is denoted by $C_{k}^{m}$. According to the cell partition method described above, nodes from every three cells do not interfere with each other.

**Figure 5:**
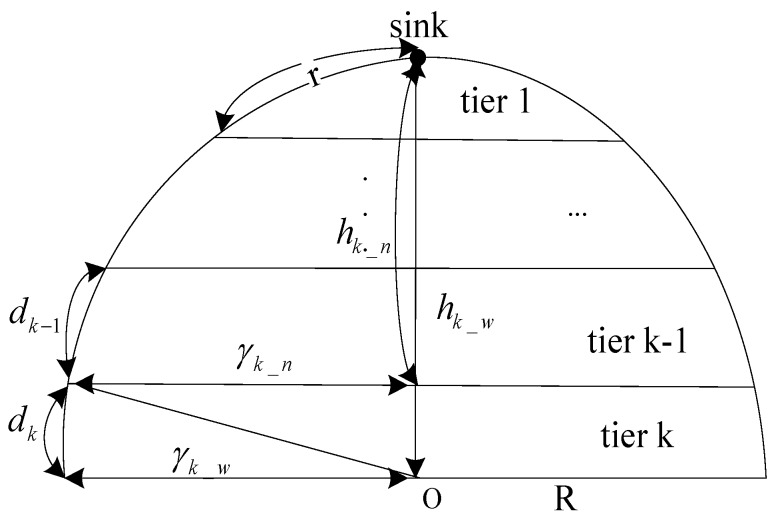
Illustration of parameters relationship for tiers and cells partition.

We use *d_i_* to denote the surface width of tier i (i=1,2,...,k), $h_{i\_w}$ and $h_{i\_n}$ respectively denote the vertebral distance from sink to the bottom transverse plane with radius of $\gamma_{i\_w}$ and to up the transverse plane with radius of $\gamma_{i\_n}$. Figure 5 illustrates the relationship among these parameters of tier $T_{k}$. As shown in Figure 5:

(4) $$\gamma_{k\_n}=R\cos\frac{d_{k}}{R}=R\cos\frac{\delta r}{R},$$

and:

(5) $$\gamma_{k\_w}=R$$

so, the number of cells in tier $T_{k}$ is:

(6) $$num(T_{k})=num_{k}=\frac{2\pi \gamma_{k\_n}}{r}=\frac{2\pi R\cos\frac{\delta r}{R}}{r}$$

Actually, it is an integer no smaller than $num_{k}$. Because:

(7) $$h_{k\_n}=R-R\sin\frac{\delta r}{R}$$

and:

(8) $$h_{k\_w}=R$$

then, we can get the area of each cell is:

(9) $$s_{cell\_k}=\frac{r}{2\pi \gamma_{k\_n}}(2\pi \gamma_{k\_w}\cdot h_{k\_w}-2\pi \gamma_{k\_n}\cdot h_{k\_n})$$

Furthermore, a simplified formula is obtained as follows:

(10) $$s_{cell\_k}=\frac{r}{\gamma_{k\_n}}(\gamma_{k\_w}\cdot h_{k\_w}-\gamma_{k\_n}\cdot h_{k\_n})$$

Finally, we can get:

(11) $$s_{cell\_k}=\frac{Rr}{\cos\frac{\delta r}{R}}(1-\cos\frac{\delta r}{R}\cdot (1-\sin\frac{\delta r}{R}))$$

Therefore, the number of nodes in each cell is $\xi_{k}=\rho \cdot s_{cell\_k}$, where ρ denotes the node density. In addition, *μ* denotes the maximum retransmission times as described in the system model. Accordingly, we can get the time slots required for collecting nodes in the outmost tier to finish data aggregation of the tier under the assumption that there is only one collecting node within each cell. The time slot is $\tau_{k\_n}$ = 3$\xi_{k}(1+\mu )$ considering the above assumption that the nodes’ interference radius is 2r so nodes from every three cells do not interfere with each other. The collecting nodes need to deliver the aggregated information to the collecting nodes in inner layer by broadcast and retransmission. Thus, the time for forwarding is $\tau_{k\_g}$ = 3(1+μ), so the total time required for all nodes in the outmost tier to finish transmission to the inner layer is computed by the following formula:

(12) $$\tau_{k}=\tau_{k\_n}+\tau_{k\_g}=3\xi_{k}(1+\mu )+3\left(1+\mu \right)$$

For the inner tier $T_{k-1}$, similar calculations can be done, so he following results can be obtained. The number of cells partitioned in the tier $T_{k-1}$ is:

(13) $$num_{k-1}=\frac{2\pi \gamma_{k-1\_n}}{r}$$

where, $\gamma_{k-1\_n}=R\cos\frac{d_{k}+d_{k-1}}{R}$. Since $\gamma_{k-1\_w}=\gamma_{k\_n}$, $h_{k-1\_w}=h_{k\_n}$, $h_{k-1\_n}=R-R\sin\frac{d_{k}+d_{k-1}}{R}$. The area of each cell is calculated by:

(14) $$\begin{array}{c} s_{cell\_k-1}=\frac{r}{2\pi \gamma_{k-1\_n}}(2\pi \gamma_{k-1\_w}\cdot h_{k-1\_w}-2\pi \gamma_{k-1\_n}\cdot h_{k-1\_n})\\ =\frac{r}{\cos\frac{d_{k}+d_{k-1}}{R}}(R\cos\frac{\delta r}{R}\cdot (1-\sin\frac{\delta r}{R})-R\cos\frac{d_{k}+d_{k-1}}{R}\cdot (1-\sin\frac{d_{k}+d_{k-1}}{R})) \end{array}$$

So, we can get the number of nodes in each cell is $\xi_{k-1}=\rho \cdot s_{cell\_k-1}$ and the time required for collecting nodes in the tier to finish data aggregation is $\tau_{k-1\_n}$ = 3$\xi_{k-1}(1+\mu )$. Considering the constraint that the tier width could meet the goal that when collecting nodes in the outer layer finish transmission to the parent collecting nodes in the inner layer and the collecting nodes in the inner layer just finish data aggregation, we have the following equation:

(15) $$\tau_{k-1\_n}=\tau_{k}$$

After simplification, it becomes:

(16) $$\xi_{k-1}=\xi_{k}+1$$

Therefore, the tier width $d_{k-1}$ can be obtained. If the procedure is repeated, we can obtain the width of each tier {$d_{k}$, $d_{k-1}$, $d_{k-2}$, …, $d_{2}$, $d_{1}$} from the outmost tier to the innermost tier under the condition $d_{k}=\delta r$ and $d_{1}=r$ which denotes the hotpots.

#### 4.2.2. Distributed In-Network Aggregation 

When the circular tier and cell partitioning is finished, collecting nodes collect the data sensed by non-collecting nodes in each cell. Besides, these collecting nodes serve the function of data aggregation and information forwarding to the sink.

**Figure 6:**
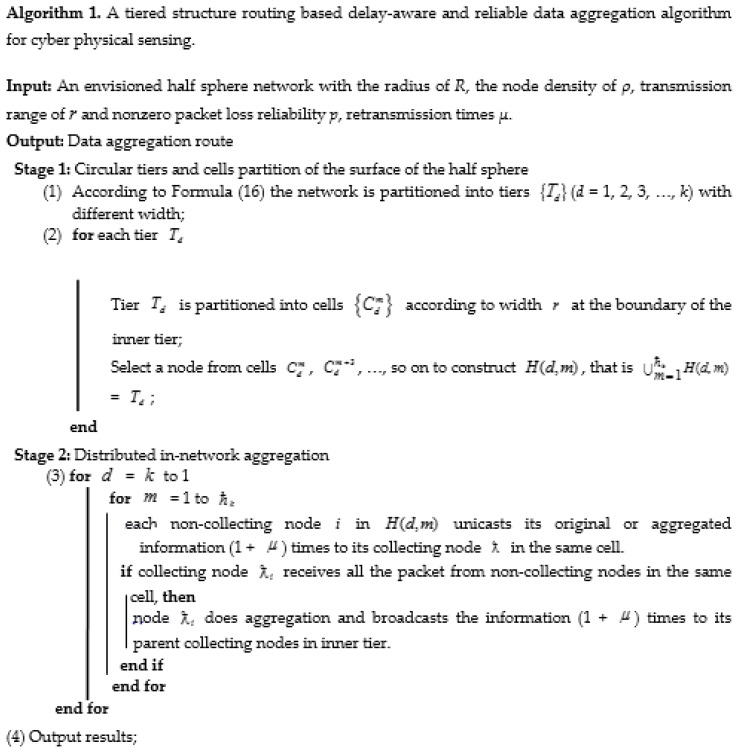
Implementation of the proposed TSR-DARDA scheme.

Assume H(k,m) denotes the subset of nodes in tier $T_{k}$ that could transmit data simultaneously, and |⋅| represents the cardinality of the set. Let one node from every three cells to be included in H(k,m), i.e., H(k,m) has a node from cells $C_{k}^{m}$, $C_{k}^{m+3}$, …, so on. Because any two nodes in H(k,m) are with distance more than 2*r*, their transmissions are without interference with each other. Furthermore, according to the cells partition in tier $T_{k}$ mentioned in the above section, all cells have the same number of nodes (possibly except one cell, which may have a smaller number of nodes), so each H(k,m) has an identical number of nodes and the number of nodes would be about a third of the number of cells, namely, it would be about a third of the number of $C_{k}^{m}$:

(17) $$\left|H(k,m)\right|=\left\lfloor \frac{1}{3}\left\lceil \frac{2\pi \gamma_{k\_n}}{r}\right\rceil \right\rfloor \;m=1,2,...,\hbar_{k}$$

where, $\hbar_{k}=\left\lceil \xi_{k}\right\rceil$. Since the wireless channels between two adjacent tiers are independent across links as described in Section 3.1, the subset of nodes *H*(*k*, *m*) in *T_k_* could transmit data simultaneously with subsets in other tiers. Thus, we could ensure simultaneous node transmission by as many as possible. When the collecting nodes in the outer layer finish transmission to the parent collecting nodes in the inner layer and the collecting nodes in the inner layer just finish data aggregation, the collecting nodes in the inner layer go on forwarding the aggregated information together to their parent collecting nodes in a more inner layer until destination sink is reached. The TSR-DARDA scheme is implemented as shown in detail in Figure 6. As shown in Figure 6, the implementation of TSR-DARDA scheme consists of two stages of circular tier, cell partitioning and distributed in-network aggregation.

**Remark.**  *The tiered structure scheme is studied in [3] combining broadcast and unicast. However, it does not consider the careful design of tier width and parallel transmission, which play an important role in delay-aware data collection. Our proposed scheme takes these issues into consideration. The VWTSR scheme proposed in [27] was aimed at flat networks. However, the proposed TSR-DARDA scheme is for spherical networks with tiered structure. The time complexity is $T(k)=O(k\cdot k)=O(k^{2})$. However, the partition of circular tiers and cells can be calculated off-line.*

### 4.3. Discussion on Parameters Selection

In the section, how to select parameters of the proposed TSR-DARDA scheme is discussed. As described in Section 4.2 above, we know that the selection of key parameters including the outmost tier width $d_{k}=\delta r$ and the number of the circular tiers plays an important role in the implementation the proposed algorithm.

#### 4.3.1. The Outmost Tier Width $d_{k}$

We know that the goal of the proposed TSR-DARDA algorithm is to enable nodes to simultaneously transmit as much data as possible under the assumption that the wireless channels between two adjacent tiers are independent across links. In order to realize the interference free transmission between two adjacent tiers, multiple transmission frequencies are required. In addition, in order to satisfy that all non-collecting nodes within each cell could transfer data packets directly to collecting nodes in the same cell, the maximum spherical distance $l_{m}$ of cell in the tier $T_{m}$ with most tier width $d_{m}$ needs to satisfy the following equation to finish the distributed data aggregation within each cell:

(18) $$l_{m}\leq ℜ$$

Under this condition, all non-collecting nodes within each cell could transfer data packets directly to collecting nodes in the same cell. According to Section 4.2.1, assume $d_{m}=f_{1}(d_{k})=f_{1}(\delta r)$. And the sum of $d_{k},d_{k-1},...,d_{m}$ is denoted by $f_{2}(\delta r)$. Besides, as shown in Figure 4, $l_{m}$ can be calculated by the following formula:

(19) $$l_{m}=R\cdot \arccos[\cos\frac{r}{R\cos\frac{f_{1}(\delta r)}{R}}\cdot \cos\frac{f_{2}(\delta r)-f_{1}(\delta r)}{R}\cdot \cos\frac{f_{2}(\delta r)}{R}+\sin\frac{f_{2}(\delta r)-f_{1}(\delta r)}{R}\cdot \sin\frac{f_{2}(\delta r)}{R}]$$

Therefore, we can get the following result:

(20) $$\arccos[\cos\frac{r}{R\cos\frac{f_{1}(\delta r)}{R}}\cdot \cos\frac{f_{2}(\delta r)-f_{1}(\delta r)}{R}\cdot \cos\frac{f_{2}(\delta r)}{R}+\sin\frac{f_{2}(\delta r)-f_{1}(\delta r)}{R}\cdot \sin\frac{f_{2}(\delta r)}{R}]\leq \frac{ℜ}{R}$$

Under the given values except δ, we can obtain the parameter *δ* range and the outmost tier width range $d_{k}=\delta r$ to solve the above inequality.

#### 4.3.2. Optimized Circular Tiers Partition

In this section, when assuming the outermost layer width $d_{k}=\delta r$, how to partition the circular tiers under the constraint of transmission range of *r* to achieve the optimization of the minimization between actual second tier width and the width in theory is discussed. 

It can be seen from the previous sections that considering the constraint that the tier width could meet the goal that when collecting nodes in the outer layer finish transmission to the parent collecting nodes in the inner layer and the collecting nodes in the inner layer just finish data aggregation, we have the equation that is $\tau_{k-1\_n}=\tau_{k}$. It is $\xi_{k-1}=\xi_{k}+1$ after simplification. Therefore, the tier width can be obtained. Thus, the second tier width ${d_{2}}^{the}$ can be theoretically calculated. However, the actual second tier width $d_{2}$ is obtained by:

(21) $$d_{2}=\frac{\pi R}{2}-\sum_{m=2}^{k}d_{m}-d_{1}$$

where, *d*_1_ represents the width of hotspots and *d*_1_ = *r*. Therefore, the optimization is to obtain the minimization of difference between the theoretically value ${d_{2}}^{the}$ and the actual value *d*_2_, which is the following formula:

(22) $$\min(\varepsilon )=\min(\left|d_{2}-d_{2}^{the}\right|)$$

**Figure 7:**
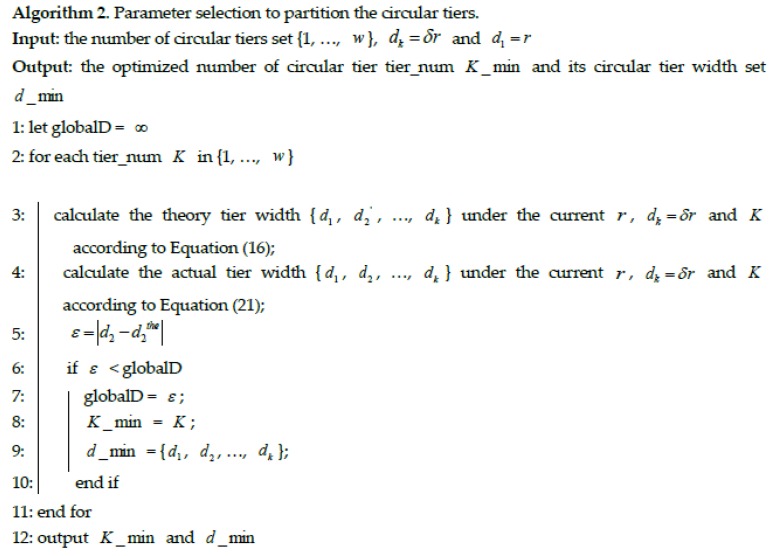
Optimization of the proposed TSR-DARDA scheme.

The minimization of difference between the theoretically value ${d_{2}}^{the}$ and the actual value $d_{2}$ can be achieved by optimizing the circular tiers partition under the constraint of transmission range of *r* and $d_{k}=\delta r$. The detailed procedure to select the optimizing parameters of the number of circular tiers is described in Figure 7. Figure 7 shows how to select parameters to optimize the proposed TSR-DARDA scheme. 

### 4.4. Performance Analysis

**Theorem** **1.** *Consider the network depth is k, Let $\hbar_{k}$ denote the total number of subsets in tier $T_{k}$. Assume the maximum number of retransmission is μ and nodes in hotspots need τ time unit to forward the aggregated information to sink. The time required for all nodes to finish a single transmission is $D=\hbar_{k}(1+\mu )+\sum_{i=1}^{k-2}\left(1+\mu \right)+\tau$.*

**Proof.**  Consider the network depth is k, which represents the number of tiers. From the Section 4.2, we know that H(k,m) is the subset of nodes in *T_k_* that can be scheduled simultaneously. Let $\hbar_{d}$ denote the total number of subsets in each tier *T_d_* such that $\cup_{m=1}^{\hbar_{d}}H(d,m)$ = $T_{d}$. Clearly, all nodes in *T_d_* can finish a single transmission in $\hbar_{d}$ time slots. Based on the assumption that nodes transmission in different tiers do not interfere with each other, the subset of nodes H(d,m) in different tiers could transmit data simultaneously. That is nodes in *T_d_* *T_d_* can start their transmissions simultaneously with nodes in outer tier $T_{d+1}$, but the number of nodes in *T_d_* is more than that in $T_{d+1}$. Assume the maximum number of retransmissions is μ. Therefore, the time needed for all nodes not in hotspots to finish data aggregation and transmission to collecting nodes in hotspots is $\hbar_{k}(1+\mu )+\sum_{i=1}^{k-2}\left(1+\mu \right)$ when the total number of tier is *k*, so, we can obtain the time required for all nodes to finish a single transmission as follows:

(23) $$D=\hbar_{k}(1+\mu )+\sum_{i=1}^{k-2}\left(1+\mu \right)+\tau □$$

**Theorem** **2.** *Let D and $D^{\Delta }$ respectively represent the time required for all nodes to finish a single transmission under the cases of network partition with the optimized TSR-DARDA scheme and other schemes. It is $D^{\Delta }-D$ > 0.*

**Proof.**  The width set are denoted by $\left\{d_{k},d_{k-1},...,d_{1}\right\}$ partitioned according to the optimized TSR-DARDA scheme from the out layer to inner layer to sink, And $\left\{d_{k},d_{k-1}^{\Delta },d_{k-2}^{\Delta }...,d_{1}\right\}$ denote the other partition case which has the identical widths of $d_{k}$ and $d_{1}=r$. □

When *k* = 4, the width set of surface circular partition are respectively {*d*_4_,*d*_3_,*d*_2_,*d*_1_} with the TSR-DARDA scheme and $\left\{d_{4},{d^{\Delta }}_{3},{d^{\Delta }}_{2},d_{1}\right\}$ without the TSR-DARDA scheme. Because the outmost tier *T*_4_ with the identical circular width under the two partition cases, the time required for nodes in the tier to finish data aggregation is identical and equals to $\hbar_{4}(1+\mu )$. For the TSR-DARDA scheme, it needs $D=\hbar_{d}(1+\mu )+(1+\mu )+(1+\mu )+\tau$ for all nodes to finish a single transmission to a sink. For other partition schemes:

(1) If ${d^{\Delta }}_{3}$ > $d_{3}$, there is ${d^{\Delta }}_{2}$ < $d_{2}$. At the time point $\hbar_{4}(1+\mu )$ + (1+μ), the tier $T_{4}$ has forwarded data aggregation to collecting nodes in tier $T_{3}$ and $T_{3}$ has not finished its own data aggregation. And more time Λ is needed to finish its own data aggregation. Therefore, all nodes to finish a single transmission to sink needs $D^{\Delta }=\hbar_{d}(1+\mu )+(1+\mu )+\Lambda +(1+\mu )+\tau$ time slots. It is obviously $D^{\Delta }-D$ > 0.
(2) If ${d^{\Delta }}_{3}$ < $d_{3}$, there is ${d^{\Delta }}_{2}$ > $d_{2}$. At the time point $\hbar_{4}(1+\mu )$ + (1+μ), $T_{3}$ has finished its own data aggregation. And, at the time point $\hbar_{4}(1+\mu )$ + (1+μ) + (1+μ), tier $T_{2}$ has not finished its own data aggregation and needs more time Γ to finish data collection to collecting nodes in the tier. Hence, the total time required to finish a single transmission for all nodes to sink is $D^{\Delta }=\hbar_{d}(1+\mu )+(1+\mu )+(1+\mu )+\Gamma +\tau$.

It is obviously $D^{\Delta }-D$ > 0. By an analogous method, when k = 5, 6, 7,…, they holds the same conclusion for other partition schemes. This proves that there is lower delay for circular partition with TSR-DARDA scheme than other partition schemes.

## 5. Performance Evaluation

In this section, the simulation results are demonstrated to evaluate the TSR-DARDA scheme. The parameter settings are described firstly, focusing more on the network model applied in simulation experiments. Then, we present the evaluation results for the proposed routing scheme. Finally, some comparisons with existing routing mechanisms are presented.

### 5.1. Parameter Settings

**Figure 8:**
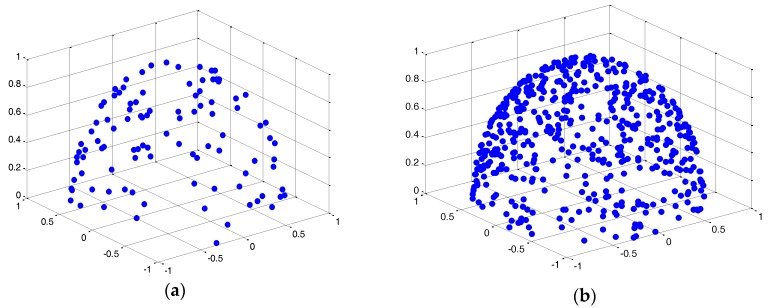
Network topology with nodes randomly scattered in half sphere surface. (**a**) 100 nodes; (**b**) 500 nodes.

The test beds are respectively two wireless sensor networks with 100 nodes and 500 nodes randomly placed on a half sphere of radius *R* = 1 (m), which are shown in Figure 8a,b. 

The sink node is located at the half sphere vertex. Let the interference range 2*r* of each node is set to 2ℂ/5, where ℂ = *πR*/2. We assume the width of the outmost tier is *δr* and *δ* = 0.6. The retransmission number *μ* is changed from 0 to 2. All links are assumed to successful transmission with the same probability *p*. Changing *p*, the number of time slots required for the sink to receive the information value is counted and the rate of packet lost is measured. Each simulation runs 1000 times and the results are averaged.

### 5.2. Evaluation on the Proposed Scheme with Different Parameters

The proposed scheme is evaluated firstly. Here, the performance evaluation mainly focuses on two metrics, namely the reliability and network delay. We investigate the performance of TSR-DARDA through simulations. When δ = 0.6, according to the implementation of the optimized TSR-DARDA shown in Figure 7, the network is divided into surface circular tiers from outer tier to inner tier. The network circular partitions for a network with 100 nodes and 500 nodes are as shown in Table 1 and Table 2, respectively, wherein $d_{1}$ represents the hotspots. Table 3 and Table 4 respectively show the number of packets lost and network delay under different *p* and *μ* values for networks with 100 nodes and with 500 nodes. As an illustration of Table 3, when *p* = 0.8 and *μ* = 0, the average number of lost packets is 13.9400 and network delay is 21.0000 time slots.

**Figure 9:**
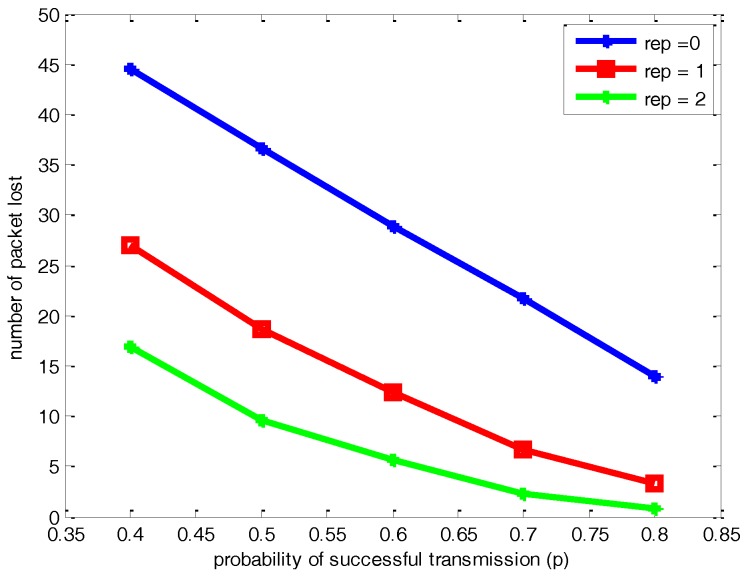
Comparisons of the number of packets lost under different *p* and *μ* for a network with 100 nodes.

**Figure 10:**
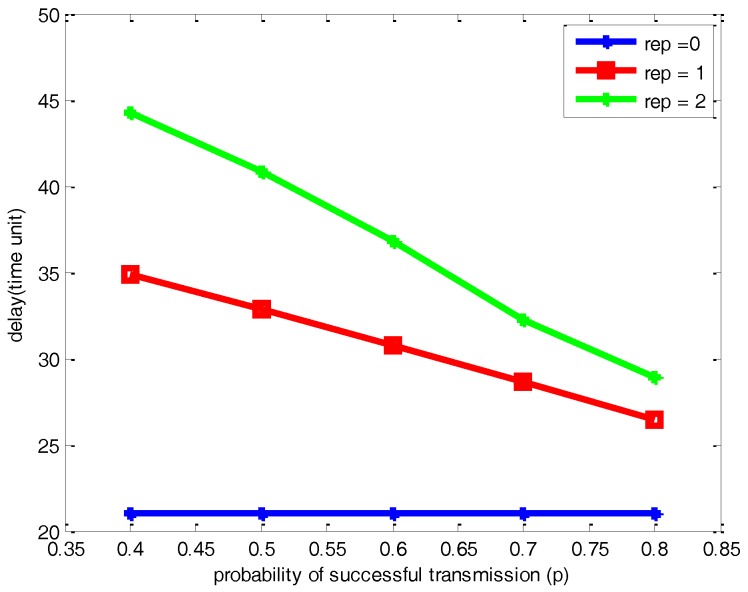
Comparisons of network delay under different *p* and *μ* for a network with 100 nodes.

Figure 9 and Figure 10 respectively demonstrate the comparison of the number of packet lost and data aggregation delay under different *p* and *μ* for a network with 100 nodes. As shown in Figure 9 and Figure 10, the changing overall trend of the number of packets lost and delay decreases as *p* becomes bigger, which is reasonable. Moreover, the number of packets lost is reduced with the increasing number of retransmission times *μ*, which leads to more data aggregation latency. When *μ* = 0, it means no retransmission of data packets in the data aggregation procedure, so there is the same network delay under different *p*.

**Figure 11:**
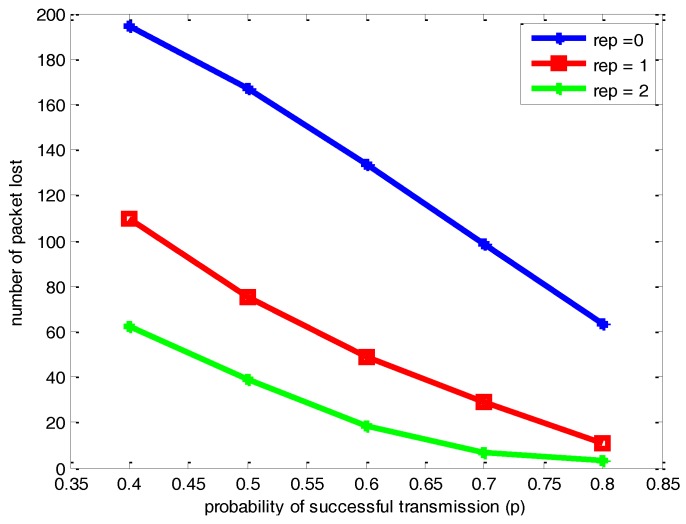
Comparisons of the number of packets lost under different *p* and *μ* for a network with 500 nodes.

**Figure 12:**
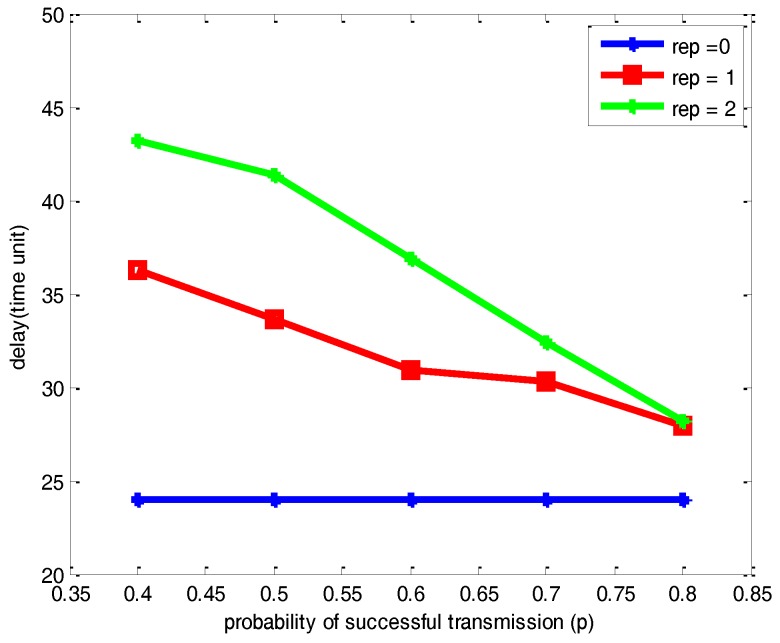
Comparisons of network delay under different *p* and *μ* for a network with 500 nodes.

Figure 11 and Figure 12 respectively illustrate the comparison of the number of packets lost and delay with different parameters of *p* and *μ* for the network with 500 nodes. As can be seen from the Figure 11 and Figure 12, it leads to the same conclusion on the changing trend of packets lost and network delay with different *p* and *μ* with a network with 100 nodes. Compared with Figure 9, Figure 11 shows that the increase of the number of nodes scattered on the half sphere of *R* = 1 leads to a certain increase of the number of packets lost with the same *p* and *μ*. However, the network reliability is not obviously changed. Comparing Figure 10 and Figure 12, there is certain increase in the network delay due to the increase of the number of nodes scattered on the half sphere.

### 5.3. Evaluation of the Proposed Scheme through Comparison

In this part, the proposed scheme is evaluated through comparisons with the tiered structure routing scheme studied in [3], wherein the circular width and network partition are not carefully designed. The comparisons focus on delay and reliability performance metrics. In order to prove the effectiveness of the proposed scheme, it is compared with the two cases of tiered structure routing without circular width and network partition carefully designed in [3]. The first compared case is named identical width tiered structure routing (IWTSR). In the case, the partitioned surface circles have the same width except the innermost and outmost layers. The second case is the surface circle width with general varied width tiered structure routing (GVWTSR). Applying the proposed scheme and the two network partition cases mentioned above, the circular tiers partition are shown in Table 5 and Table 6 for networks with nodes 100 and 500, respectively. 

**Figure 13:**
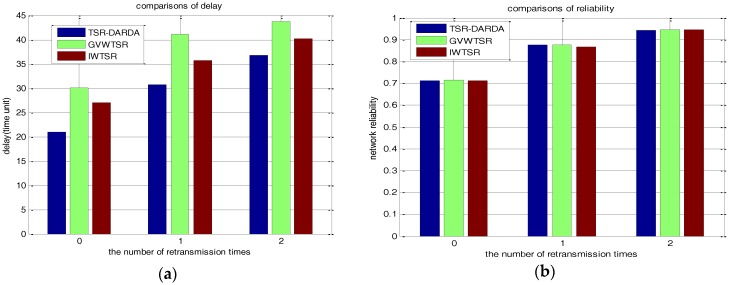
Comparison under different *μ* for a network with 100 nodes when *p* = 0.6. (**a**) The network delay; (**b**) The number of packets lost.

**Figure 14:**
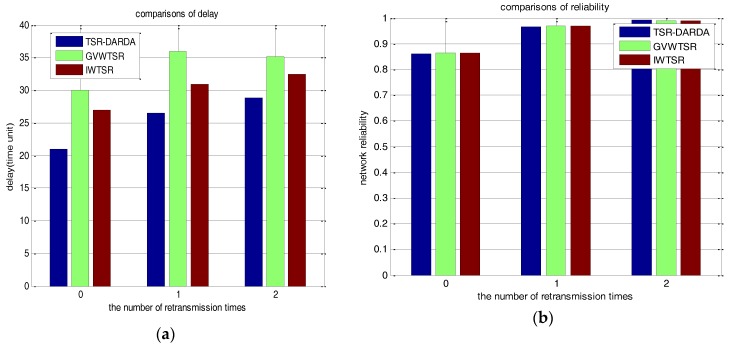
Comparison under different *μ* for a network with 100 nodes when *p* = 0.8. (**a**) The network delay; (**b**) The number of packets lost.

Comparing TSR-DARDA with IWTSR and GVWTSR under the abovementioned circular tiers partition for network with 100 nodes, we randomly selected two cases of *p* = 0.6 and *p* = 0.8 under different *μ* to compare the number of packets lost and network delay. The comparison results are respectively shown in Table 7 and Table 8. For the sake of intuition, Figure 13a,b respectively show the corresponding comparisons on the metrics of the number of packet lost and network delay by exploiting the different schemes of TSR-DARDA, IWTSR and GVWTSR when *p* = 0.6. As shown in Figure 13a, the proposed TSR-DARDA is superior in delay though the network reliability changes not obviously as shown in Figure 13b. When μ = 0, the network delay is respectively improved by 22.22% and 30.00% compared with IWTSR and GVWTSR. It can be seen from the Figure 14 that it holds the same conclusion with Figure 13. This demonstrates that the proposed TSR-DARDA could effectively minimize data collection latency with the assurance of network reliability.

**Figure 15:**
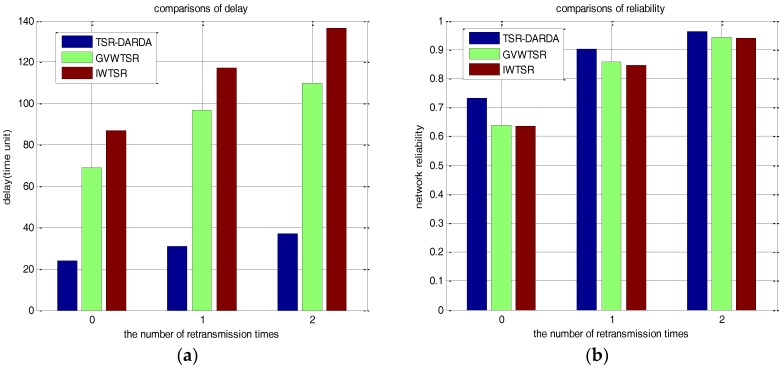
Comparison under different *μ* for a network with 500 nodes when *p* = 0.6. (**a**) The network delay; (**b**) The number of packets lost.

For the three cases of applying TSR-DARDA, IWTSR and GVWTSR schemes for the network with 500 nodes divided into circular tiers with width as shown in Table 6, the comparisons of the number of packets lost and network delay under randomly selected *p* = 0.6 and different *μ* are shown in Table 9. Figure 15a,b intuitively and respectively show the corresponding comparisons on the metrics of the number of packets lost and network delay by exploiting the different schemes of TSR-DARDA, IWTSR and GVWTSR. As shown in Figure 15a,b, the proposed TSR-DARDA is superior both in reliability and delay than the other two schemes when *p* = 0.6 for a half spherical network with 500 nodes, which further demonstrates that the proposed TSR-DARDA could effectively reduce data collection delay with satisfactory network reliability. Also, we could see that the advantage on network delay is more obvious with the increasing number of nodes scattered on the half sphere.

**Figure 16:**
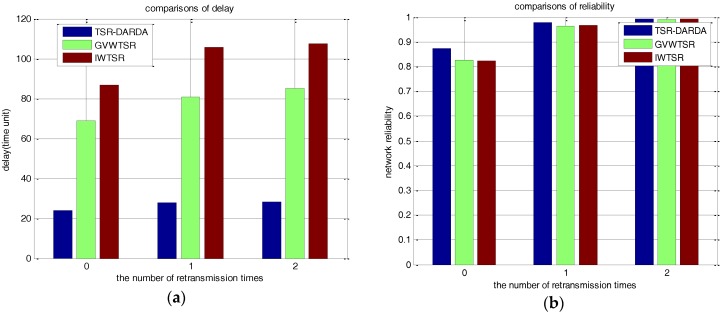
Comparison under different *μ* for a network with 500 nodes when *p* = 0.8. (**a**) The network delay; (**b**) The number of packets lost.

When the randomly selected *p* = 0.8, the comparisons of the number of packet lost and network delay under different *μ* are shown in Table 10. Figure 16 intuitively shows the results. As can be seen from the figure, the same conclusion as the case of *p* = 0.6 holds. This further proves the effectiveness of TSR-DARDA, which could achieve performance improvement in transport delay with satisfactory network reliability.

## 6. Conclusions

In this paper, the problem of an effective data collection scheme to reduce network delay with tiered structure routing for spherical objects sensing is studied. Data collection should take reliability as well as delay into consideration. To address these problems, a novel routing scheme named TSR-DARDA is proposed. Carefully designed system parameters grant TSR-DARDA lower transport delay under given reliability constraints. The theoretical analysis shows that the method outperforms the existing methods in network delay. In addition, it can be seen from the simulation results that the network delay could be reduced while guaranteeing reliability. 

However, the characteristics of wireless communications are not considered fully, including the abundance of physical obstacles, multipath propagation and electromagnetic interference from equipment and coexisting wireless systems, etc. Besides, there are many interesting open questions to consider. Although we focus on the delay performance, other performance metrics such as time complexity and the communication overhead of the routing protocol are also of importance. It would be interesting to study the relationship between these metrics with data aggregation and network topologies.

## Figures and Tables

**Table 1:** Circular tiers partition for networks with 100 nodes.

Tier	Width (m)
*d*_1_	0.3142
*d*_2_	0.6273
*d*_3_	0.4408
*d*_4_	0.1885

**Table 2:** Circular tiers partition for networks with 500 nodes.

Tier	Width (m)
*d*_1_	0.3142
*d*_2_	0.2884
*d*_3_	0.4578
*d*_4_	0.3220
*d*_5_	0.1885

**Table 3:** The number of packets lost and network delay under different *p* and *μ* for a network with 100 nodes.

Number of Packets Lost/Delayed	*p*				
*μ*	*p* = 0.4	*p* = 0.5	*p* = 0.6	*p* = 0.7	*p* = 0.8
*μ* = 0	44.4900/21.0000	36.5600/21.0000	28.9100/21.0000	21.7000/21.0000	13.9400/21.0000
*μ* = 1	26.9300/34.8600	18.6500/32.8800	12.3700/30.7200	6.6100/28.6800	3.2200/26.4900
*μ* = 2	16.8300/44.2800	9.5500/40.8600	5.6500/36.7800	2.3000/32.2800	0.8500/28.8600

**Table 4:** The number of packets lost and network delay under different *p* and *μ* for a network with 500 nodes.

Number of Packets Lost/Delayed	*p*				
*μ*	*p* = 0.4	*p* = 0.5	*p* = 0.6	*p* = 0.7	*p* = 0.8
*μ* =0	194.5000/24.0000	166.7000/24.0000	133.8000/24.0000	98.5000/24.0000	63.5000/24.0000
*μ* =1	109.8000/36.3000	74.9000/33.6000	49.1000/30.9000	29.2000/30.3000	10.9000/27.9000
*μ* =2	62.0000/43.2000	38.9000/41.4000	18.7000/36.9000	6.9000/32.4000	3.4000/28.2000

**Table 5:** Different surface circular tiers partition for network with 100 nodes.

Width	TSR-DARDA	IWTSR	GVWTSR
*d*_1_	0.3142	0.3142	0.3142
*d*_2_	0.6273	0.5341	0.5682
*d*_3_	0.4408	0.5341	0.5000
*d*_4_	0.1885	0.1885	0.1885

**Table 6:** Different surface circular tiers partition for network with 500 nodes.

Width	TSR-DARDA	IWTSR	GVWTSR
*d*_1_	0.3142	0.3142	0.3142
*d*_2_	0.2884	0.3561	0.4682
*d*_3_	0.4578	0.3561	0.4000
*d*_4_	0.3220	0.3561	0.2000
*d*_5_	0.1885	0.1885	0.1885

**Table 7:** Comparison of the number of packets lost and network delay under different *μ* for a network with 100 nodes when *p* = 0.6.

Number of Packets Lost/Delayed *p* = 0.6	TSR-DARDA	IWTSR	GVWTSR
*μ* = 0	28.9100/21.0000	28.5000/30.0000	28.9000/27.0000
*μ* = 1	12.3700/30.7200	12.4000/41.1000	13.4000/35.7000
*μ* = 2	5.6500/36.7800	5.4000/43.8000	5.4000/40.2000

**Table 8:** Comparison of the number of packets lost and network delay under different *μ* for a network with 100 nodes when *p* = 0.8.

Number of Packets Lost/Delayed *p* = 0.8	TSR-DARDA	IWTSR	GVWTSR
*μ* = 0	13.9400/21.0000	13.5000/30.0000	13.6000/27.0000
*μ* = 1	3.2200/26.4900	3.0000/36.0000	3.0000/30.9000
*μ* = 2	0.8500/28.8600	1.0000/35.1000	0.9000/32.4000

**Table 9:** Comparison of the number of packets lost and network delay under different *μ* for a network with 500 nodes when *p* = 0.6.

Number of Packets Lost/Delayed *p* = 0.6	TSR-DARDA	IWTSR	GVWTSR
*μ* = 0	133.8000/24.0000	180.2000/69.0000	181.4000/87.0000
*μ* = 1	49.1000/30.9000	70.6000/96.9000	76.3000/117.3000
*μ* = 2	18.7000/36.9000	29.2000/109.8000	30.7000/136.5000

**Table 10:** Comparison of the number of packets lost and network delay under different *μ* for a network with 500 nodes when *p* = 0.8.

Number of Packets Lost/Delayed *p* = 0.8	TSR-DARDA	IWTSR	GVWTSR
*μ* = 0	63.5000/24.0000	87.3000/69.0000	88.2000/87.0000
*μ* = 1	10.9000/27.9000	18.1000/81.0000	16.1000/105.9000
*μ* = 2	3.4000/28.2000	4.4000/85.1000	4.1000/107.4000

## References

[1] Ergen S.C., Varaiya P. (2010). TDMA Scheduling Algorithms for Wireless Sensor Networks. Wirel. Netw..

[2] Dong M.X., Ota K., Liu A.F. (2016). RMER: Reliable and Energy Efficient Data Collection for Large-scale Wireless Sensor Networks. IEEE Internet Things J..

[3] Joo C., Shroff N.B. (2014). On the Delay Performance of In-Network Aggregation in Lossy Wireless Sensor Networks. IEEE/ACM Trans. Netw..

[4] Zhang Q., Liu A.F. (2016). An Unequal Redundancy Level Based Mechanism for Reliable Data Collection in Wireless Sensor Networks. EURASIP J. Wirel. Commun. Netw..

[5] Akan O.B., Akyildiz I.F. (2005). Event-to-sink Reliable Transport in Wireless Sensor Networks. IEEE/ACM Trans. Netw..

[6] Kim R.E., Mechitov K., Sim S.H., Spencer B.F., Song J.H. (2016). Probabilistic Assessment of High-Throughput Wireless Sensor Networks. Sensors.

[7] Ibayashi H., Kaneda Y., Imahara J., Oishi N., Kuroda M., Mineno H. (2016). A Reliable Wireless Control System for Tomato Hydroponics. Sensors.

[8] Dong M.X., Ota K., Yang L.T., Liu A.F., Guo M.Y. (2016). LSCD: A Low Storage Clone Detecting Protocol for Cyber-Physical Systems. IEEE Trans. Comput.-Aided Des. Integr. Circuits Syst..

[9] Liu Y.H., Zhu Y.M., Ni L.M., Xue G.T. (2011). A Reliability-Oriented Transmission Service in Wireless Sensor Networks. IEEE Trans. Parallel Distrib. Syst..

[10] Liu X., Dong M.X., Ota K., Hung P., Liu A.F. (2016). Service Pricing Decision in Cyber-Physical Systems: Insights from Game Theory. IEEE Trans. Serv. Comput..

[11] Claycomb W.R., Shin D. (2011). A Novel Node Level Security Policy Framework for Wireless Sensor Networks. J. Netw. Comput. Appl..

[12] Shu T., Krunz M., Liu S.S. (2011). Secure Data Collection in Wireless Sensor Networks Using Randomized Dispersive Routes. IEEE Trans. Mobile Comput..

[13] Yousefi H., Mizanian K., Jahangir A.H. Modeling and Evaluating the Reliability of Cluster Based Wireless Sensor Networks. Proceedings of the 24th IEEE International Conference on Advanced Information Networking and Applications (AINA).

[14] Lu C.Y., Saifullah A., Li B., Sha M., Gonzalez H., Gunatilaka D., Wu C., Nie L., Chen Y.X. (2016). Real-Time Wireless Sensor-Actuator Networks for Industrial Cyber-Physical Systems. Proc. IEEE.

[15] Gungor V.C., Akan O.B. (2007). Delay Aware Reliable Transport in Wireless Sensor Networks. Int. J. Commun. Syst..

[16] Angelopoulos C.M., Nikoletseas S., Patroumpa D., Raptopoulos C. (2016). Efficient Collection of Sensor Data via a New Accelerated Random Walk. Concurr. Comput.-Pract. Exp..

[17] Hu Y.L., Dong M.X., Ota K., Liu A., Guo M. (2016). Mobile Target Detection in Wireless Sensor Networks with Adjustable Sensing Frequency. IEEE Syst. J..

[18] Lin H.F., Bai D., Gao D.M., Liu Y.F. (2016). Maximum Data Collection Rate Routing Protocol Based on Topology Control for Rechargeable Wireless Sensor Networks. Sensors.

[19] Long J., Liu A.F., Dong M.X., Li Z. (2015). An Energy-Efficient and Sink-Location Privacy Enhanced Scheme for WSNs through Ring based Routing. J. Parallel Distrib. Comput..

[20] Chen Z.B., Liu A.F., Li Z.T., Choi Y., Sekiya H., Li J. (2017). Energy-efficient Broadcasting Scheme for Smart Industrial Wireless Sensor Networks. Mob. Inf. Syst..

[21] Li D.Y., Zhu Q.H., Zhu Y.Q., Du H.W., Wu W.L. (2015). Conflict-Free Many-to-One Data Aggregation in Multi-Channel Multi-Hop Wireless Networks. Int. J. Sens. Netw..

[22] Li X.Y., Wang Y.J., Wang Y. (2011). Complexity of Data Collection, Aggregation, and Selection for Wireless Sensor Networks. IEEE Trans. Comput..

[23] Li T., Liu A.F., Huang C.Q. (2016). A Similarity Scenario-based Recommendation Model with Small Disturbances for Unknown Items in Social Networks. IEEE Access.

[24] Shanti C., Sahoo A. (2010). DGRAM: A Delay Guaranteed Routing and MAC Protocol for Wireless Sensor Networks. IEEE Trans. Mobile Comput..

[25] Rosberg Z., Liu R.P., Dinh T.L., Dong Y.F., Jha S. (2010). Statistical Reliability for Energy Efficient Data Transport in Wireless Sensor Networks. Wirel. Netw..

[26] Akkari W., Bouhdid B., Belghith A. LEATCH: Low Energy Adaptive Tier Clustering Hierarchy. Proceedings of 6th International Conference on Ambient Systems, Networks and Technologies (ANT)/5th International Conference on Sustainable Energy Information Technology (SEIT).

[27] Zhang J.H., Long J., Zhao G.H., Zhang H. (2015). Minimized Delay with Reliability Guaranteed by Using Variable Width Tiered Structure Routing in WSNs. Int. J. Distrib. Sens. Netw..

[28] Suto K., Nishiyama H., Kato N., Huang C.W. (2015). An Energy-Efficient and Delay-Aware Wireless Computing System for Industrial Wireless Sensor Networks. IEEE Access.

